# Structure of human steroid 5α-reductase 2 with anti-androgen drug finasteride

**DOI:** 10.21203/rs.3.rs-40159/v1

**Published:** 2020-07-15

**Authors:** Qingpin Xiao, Lei Wang, Shreyas Supekar, Tao Shen, Heng Liu, Fei Ye, Junzhou Huang, Hao Fan, Zhiyi Wei, Cheng Zhang

**Affiliations:** 1 Department of Biology, Southern University of Science and Technology, Shenzhen, Guangdong 518055, China; 2 Department of Pharmacology and Chemical Biology, School of Medicine, University of Pittsburgh, Pittsburgh, PA15261, USA; 3 Bioinformatics Institute (BII), Agency for Science, Technology and Research (A*STAR), Singapore 138671, Singapore; 4 Tencent AI Lab, Shenzhen, Guangdong 518000, China; 5 Faculty of Health Sciences, University of Macau, Macau SAR 999078, China.

## Abstract

Human steroid 5α-reductase 2 (SRD5α2) as a critical integral membrane enzyme in steroid metabolism catalyzes testosterone to dihydrotestosterone. Mutations on its gene have been linked to 5α-reductase deficiency and prostate cancer. Finasteride and dutasteride as SRD5α2 inhibitors are widely used anti-androgen drugs for benign prostate hyperplasia, which have recently been indicated in the treatment of COVID-19. The molecular mechanisms underlying enzyme catalysis and inhibition remained elusive for SRD5α2 and other eukaryotic integral membrane steroid reductases due to a lack of structural information. Here, we report a crystal structure of human SRD5α2 at 2.8 Å revealing a unique 7-TM structural topology and an intermediate adduct of finasteride and NADPH as NADP-dihydrofinasteride in a largely enclosed binding cavity inside the membrane. Structural analysis together with computational and mutagenesis studies reveals molecular mechanisms for the 5α-reduction of testosterone and the finasteride inhibition involving residues E57 and Y91. Molecular dynamics simulation results indicate high conformational dynamics of the cytosolic region regulating the NADPH/NADP^+^ exchange. Mapping disease-causing mutations of SRD5α2 to our structure suggests molecular mechanisms for their pathological effects. Our results offer critical structural insights into the function of integral membrane steroid reductases and will facilitate drug development.

The membrane-embedded 5α-reductase family in the human includes five members, SRD5α1-3 and much less characterized glycoprotein synaptic 2 (GSPN2) and GSPN2-like^1^. They mainly catalyze the irreversible reduction of the Δ^4,5^ bond in Δ^4^-3-ketosteroids using reduced nicotinamide adenine dinucleotide phosphate (NADPH) as the hydride donor cofactor, though other lipid substrates have also been identified^1-3^. SRD5αs are expressed differently in human body to play diverse functional roles despite their sequence similarity (Extended Data Fig. 1). SRD5α1 and SRD5α3 have been indicated to function in the metabolism of neurosteroids^4,5^ and the protein *N*-linked glycosylation^2,6^, respectively. SRD5α2 is the most intensively investigated SRD5α with well-characterized roles in androgen metabolism and androgen-related disorders^1,7^. All three SRD5αs are located in the membrane of endoplasmic reticulum (ER) in the cells^8^.

SRD5α2 is highly expressed in male reproductive systems^7^ to convert testosterone to 5α-dihydrotestosterone (DHT) (Fig. 1a), the major steroid hormone for androgen receptor^1^. A large number of mutations identified in the *SRD5A2* gene can result in inefficient levels of DHT, leading to an autosomal recessive disorder named 5α-reductase deficiency associated with underdeveloped and atypical genitalia^9-11^. On the other hand, overproduction of DHT by SRD5α2 is associated with benign prostatic hyperplasia (BPH), androgenic alopecia and prostate cancer due to excessive androgen receptor signaling^7,12^. 5α-reductase inhibitors (5ARIs) including finasteride and dutasteride (Fig. 1b), which mainly target SRD5α2 but also act on other SRD5αs^13^, have been used as a major anti-androgenic class of drugs to treat BPH and androgenic alopecia^1,7,12,14^ and indicated in the treatment of prostate cancer^15^. In particular, finasteride is among the top-100 most-prescribed drugs in the United States, which is associated with an irreversible action on SRD5α2^16,17^. Interestingly, androgen receptor signaling has recently been linked to COVID-19 disease severity, explaining why males are more prone to severe COVID-19 symptoms^18^. The 5ARI drugs have thus been suggested to hold the repurposing potential for treating COVID-19^18,19^.

SRD5αs belong to a large group of eukaryotic membrane-embedded steroid reductases, which also include sterol reductases such as the 7-dehydrocholesterol reductase (DHCR7) that catalyzes the last step in cholesterol biosynthesis in humans^20^. Although these steroid/sterol reductases share very little sequence similarity, they all use NADPH as the cofactor to reduce specific carbon-carbon double bonds in their steroid substrates. So far, only one crystal structure of a bacterial membrane-embedded sterol reductase MaSR1 without any steroid substrate was reported for this group of reductases^21^. To further understand the molecular mechanisms underlying the function of eukaryotic steroid reductases and in particular the catalytic mechanism of SRD5αs and the action of 5ARI drugs, we solved a crystal structure of human SRD5α2 in the presence of NADPH and finasteride. The structure revealed a topology of 7 transmembrane α-helices (7 TMs), rather than the 10-TM topology of MaSR1, and an NADP-dihydrofinasteride intermediate adduct. The structure together with computational studies provided unprecedented molecular insights into the catalytic mechanism of SRD5α2, the irreversible action of finasteride on SRD5α2, and the molecular mechanisms underlying the pathological effects of disease-associated mutations.

## Results

### Structure determination and overall structure of human SRD5α2

**Fig. 1:**
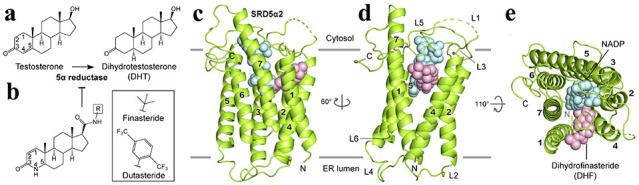
Overall structure of human SRD5α2. **(a**) 5α-reduction reaction of the Δ^4,5^ double bond of testosterone catalyzed by SRD5α2 to generate dihydrotestosterone (DHT). (**b)** SRD5α2 inhibition by finasteride and dutasteride. The two inhibitors share the same ring structure with different R-groups connected in the tails. **(c)-(e)** Three views of the SRD5α2 structure. The NADP-DHF adduct was shown as spheres. L1-6 represent 6 loops connecting 7-TMs. The NADP and DHF moieties were colored in light cyan and light pink, respectively.

Human SRD5α2 was expressed in insect Sf9 cells and purified in the presence of finasteride and crystallized in lipidic mesophase with a space group of *P622* (Extended Data Fig. 2)^22^. The structure was determined to 2.8-Å resolution by molecular replacement^23^ based on a structural model from *de novo* prediction since we failed to use anomalous diffraction data to solve the structure (see Methods). Clear electron density allowed modeling of all 254 residues of SRD5α2 except for the first 4 residues and residues S39-A43 in a flexible loop region (Fig. 1c, d and Table 1). A dualsteric ligand was modeled as an adduct of finasteride and NADPH in the structure (Fig. 1e), which will be discussed later.

Unlike the bacterial sterol reductase MaSR1^21^, the structure of SRD5α2 contains 7 TMs (TM1-7) connected by 6 loops (L1-6) (Fig. 1c, d and Extended Data Fig. 3a). We assigned the carboxyl terminal (C-terminal) loop (C-loop) to face the cytosol and the amino terminal loop (N-loop) to face ER lumen according to the enriched positively charged residues at the C-terminal side^24^ (Extended Data Fig. 3b). In addition, the N-terminal residue C5^N^ (superscripts indicate location of the residues hereafter) forms a disulfide bond with C133^L4^ in loop 4 (L4) (Extended Data Fig. 3a), suggesting an ER luminal location of the N-terminal side because of the reducing environment of the cytosol. The 7-TM topology is more commonly associated with G protein-coupled receptors (GPCRs)^25^, although the arrangement of TMs in SRD5α2 is distinct from that of GPCRs (Extended Data Fig. 3c).

### Intermediate adduct formed between finasteride and NADPH

**Fig. 2:**
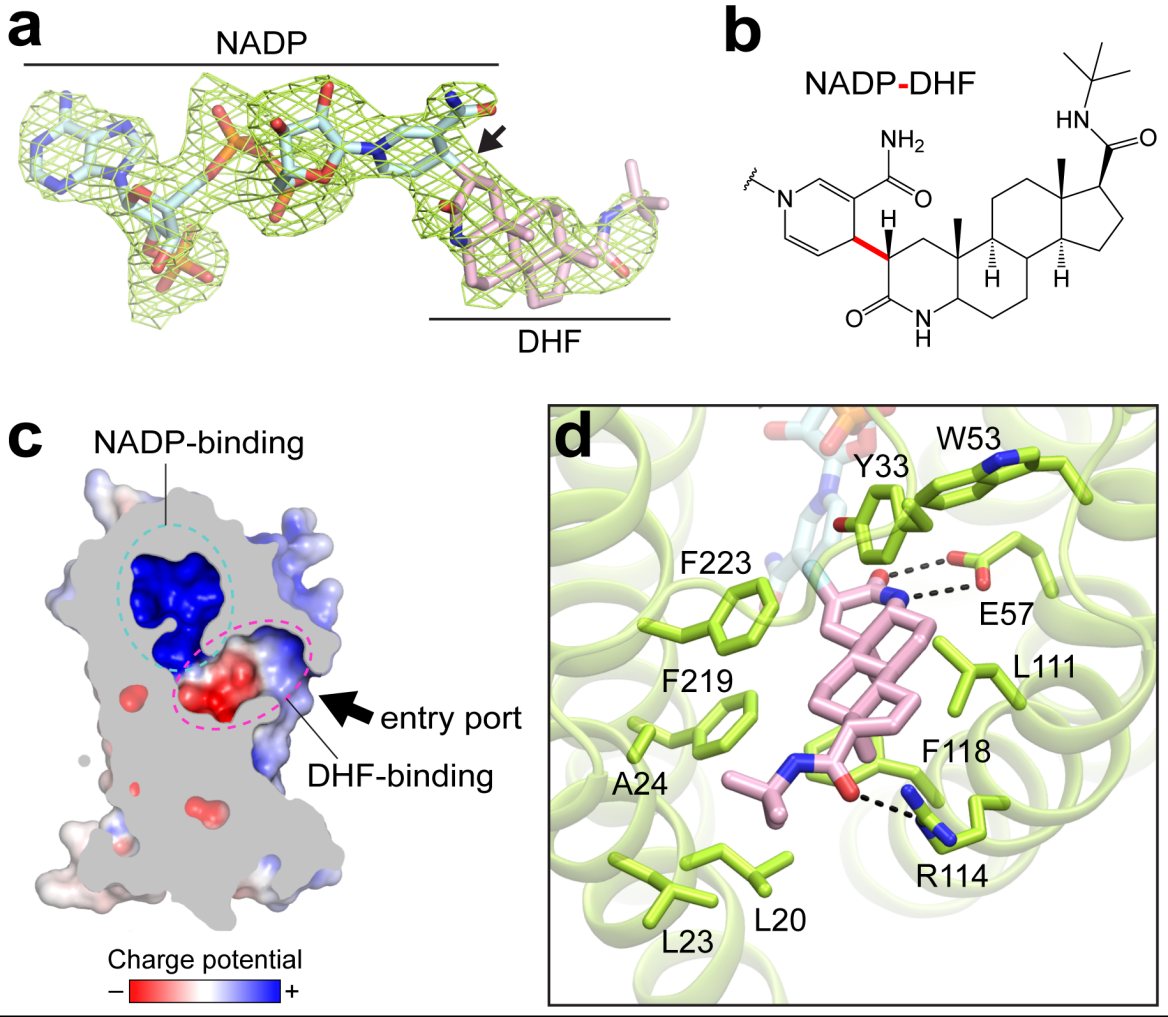
Formation and binding environment of NADP-DHF adduct. **(a)** *F_o_-F_c_* electron omit map of the NADP-DHF adduct contoured at 3σ. **(b)** Chemical structure of the NADP-DHF adduct. The covalent bond connecting the nicotinamide C-4 atom of NADPH and the C-2 atom of finasteride was highlighted in red. **(c)** Enclosed binding cavity for NADP-DHF with charge potentials. The potential entry port for the steroid substrates was indicated by an arrow. **(d)** Molecular details of the DHF binding pocket. Hydrogen bonds were indicated by dashed lines.

The structure of SRD5α2 revealed a large cavity inside the 7-TM domain at the cytosolic side formed by all 7 TMs and L1, L3 and L5 (Fig. 1c, d). The cavity is completely occluded from the cytosol with only one opening on the side of 7-TM domain between TM1 and TM4. Clear electron density in the cavity revealed features of NADPH and finasteride, which allowed an unambiguous modeling of both ligands (Fig. 2a). Strikingly, after ligand fitting, the distance between the nicotinamide C-4 atom of NADPH and the C-2 atom of finasteride is shorter than 2Å, suggesting the formation of a covalent bond (Fig. 2a, b).

Strongly supporting our structural findings, a previous enzymological and mass spectrometric study on the mechanism of inhibition of SRD5α2 by finasteride identified an intermediate adduct as NADP-dihydrofinasteride (NADP-DHF)^17^. The same study indicated that SRD5α2 could catalyze the hydride transfer from NADPH to finasteride, leading to the formation of a covalent bond between the nicotinamide C-4 atom of NADPH and the C-2 atom of finasteride^17^, highly consistent with our structural observation. It is likely that SRD5α2 took endogenous NADPH during recombinant protein expression to catalyze the reaction with finasteride supplemented in protein buffers to generate the NADP-DHF intermediate, which was stable enough to be captured in our SRD5α2 crystals. In fact, NADP-DHF has been suggested to be one of the most potent non-covalent enzyme inhibitors in general with a very slow dissociation rate, explaining the irreversible action of finasteride on SRD5α2^13^. Therefore, we modeled NADP-DHF as the real ligand in our structure.

### Binding pockets for NADP-DHF

The substrate-binding cavity shows two relatively separate tunnel-like pockets for NADP and DHF (Fig. 2c). NADP adopts an extended anti-conformation to insert into the binding pocket with a positively charged environment inside the 7-TM bundle (Fig. 2c). Surprisingly, the binding pocket for NADP is enclosed at the cytosolic side by the cytosolic loops, completely shielding NADP from the cytosol (Fig. 1e). The nicotinamide-ribose moiety is buried inside 7-TM and stabilized by extensive polar and hydrophobic interactions with residues mainly from TMs 2, 4-7, while the diphosphate moiety of NADP mainly forms polar interactions with L1 and TM6-7 (Extended Data Fig. 4a-e). The adenine-ribose phosphate moiety of NADP forms hydrogen bonds and salt bridges with residues mostly from three cytosolic loops (Extended Data Fig. 4a-e). Supporting such a cofactor-binding mode, mutations of residues that interact with NADP have been shown to either decrease or abolish the catalytic activity of SRD5α2 by previous enzymological studies (Extended Data Fig. 4d)^26-28^.

In contrast to the highly polar environment for NADP, the binding pocket of DHF is largely hydrophobic, where the core ring structure of DHF interacts with hydrophobic residues from TMs 1, 2, 4 and 7 (Fig. 2d and Extended Data Fig. 4e). A previous study showed that substitution of one aromatic residue F118^TM4^ in the DHF-binding pocket to a leucine could dramatically decrease the SRD5α2 activity by disrupting the binding of testosterone^29^, suggesting an important role of this residue in the binding of steroid substrates. The polar groups located at each end of DHF engage in additional polar interactions with residues E57^TM2^ and R114^TM4^ of SRD5α2 (Fig. 2d).

Sequence alignment analysis of SRD5αs across different species including a plant homologue DET2 involved in the synthesis of phytohormones^30^ indicated that while the residues involved in the binding of NADP are highly conserved, the residues in the DHF-binding pocket are much less conserved (Extended Data Fig. 1). This suggests that although all the SRD5α family members use NADPH as the cofactor to reduce their substrates, they have evolved specific structural features to recognize different steroid or lipid substrates^1,7^. We further mapped the residue conservation to the SRD5α2 structure, which showed that the region around the tert-butylacetamide tail group of DHF is the least conserved part of the ligand-binding pocket (Extended Data Fig. 4f), explaining the selectivity of finasteride for human SRD5α1 over SRD5α2^1,14^.

### Potential mechanisms of SRD5α2 catalysis and inhibition

**Fig. 3:**
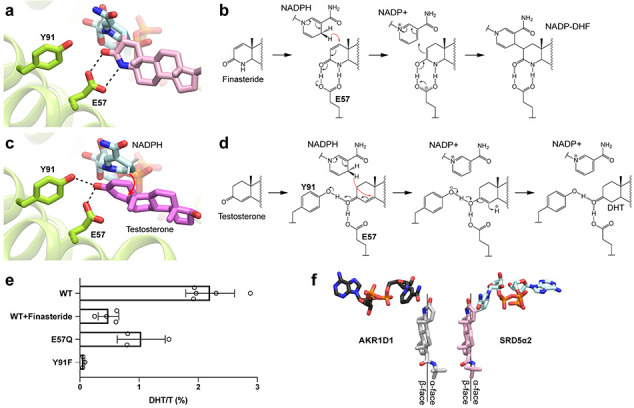
Mechanisms for SRD5α2 catalysis and inhibition. **(a)** Binding pose of DHF. Y91^TM3^ is not directly hydrogen bonding to DHF. **(b)** Potential mechanism for finasteride inhibition and the covalent adduct formation between NADPH and finasteride. E57^TM2^ facilitates the hydride transfer to the Δ^1,2^ bond of finasteride, leading to the formation of a covalent bond. **(c)** Binding pose of testosterone based on our docking results. E57^TM2^ and Y91^TM3^ each forms a hydrogen bond with the substrate. **(d)** Potential mechanism the 5α-reduction of testosterone. E57^TM2^ and Y91^TM3^ facilitate the hydride transfer to the Δ^4,5^ bond of testosterone, leading to the formation of DHT. Hydrogen bonds are shown as dashed lines and the hydride transfer is shown as red curved arrows. **(e)** Catalysis of testosterone (T) to dihydrotestosterone (DHT) by wild type SRD5α2 (WT), WT with 500 μM finasteride and two SRD5α2 mutants E57Q and Y91F. The ratios of DHT to T (DHT/T) were determined by mass spectrometry. All data are presented as mean ± SEM of 3-5 independent experiments. **(f)** Distinct orientations of finasteride relative to NADPH in AKR1D1 and SRD5α2. The finasteride and NADPH conformations in the AKR1D1 structure (PDB ID 3G1R) were shown by align the core ring of finasteride to that of DHF in the SRD5α2 structure.

It has been proposed that two unknown residues in SRD5α2 point toward the C-3 carbonyl group in finasteride to facilitate the hydride transfer from NADPH to finasteride to form an enolized intermediate, which is followed by the formation of a covalent bond between finasteride and NADP^17^. In our structure, both the C-3 carbonyl and the N-4 amine groups of DHF form hydrogen bonds with E57^TM2^ (Fig. 3a). We propose that through hydrogen-bonding interactions with E57^TM2^, finasteride is positioned in a way that the 4-pro-(*R*)-hydride of NADPH is in the proximity of the C-2 atom of finasteride to allow the hydride transfer to the Δ^1,2^ bond in finasteride. Due to the presence of the N-4 amine group, the enolization of finasteride as the result of hydride transfer involves the C-3 carbonyl and the C-2 group, leading to the covalent bond formation between finasteride and NADP (Fig. 3b). Dutasteride contains the same core ring structure as finasteride (Fig. 1b) and therefore likely forms a similar adduct with NADPH. Indeed, dutasteride has been suggested to share the same irreversible inhibition mechanism as finasteride^31^.

Interestingly, finasteride also inhibits the activity of steroid 5β-reductase, a soluble steroid reductase that belongs to the NADPH-dependent aldo-keto reductase (AKR) superfamily^32^. Steroid 5β-reductase, also named AKR1D1, can reduce the Δ^4,5^ bond in testosterone as SRD5α2 but to generate a stereochemically different product, 5β-DHT^3^. A crystal structure of AKR1D1 with finasteride ^33^ showed that the relative position of finasteride to NADP^+^ in AKR1D1 is opposite to that of DHF to NADP in SRD5α2 (Extended Data Fig. 4g). As a result, the 4-pro-(*R*)-hydride of NADPH is adjacent to the N-4 group instead of the C-2 group of finasteride (Extended Data Fig. 4g), thereby preventing hydride transfer^33^. Hence, the Δ^1^,^2^ bond of finasteride cannot be reduced by AKR1D1, which accounts for the competitive and reversible action of finasteride on AKR1D1^33^.

To investigate the binding pose of testosterone and the catalytic mechanism of SRD5α2, we docked NADPH and testosterone to our structure *in silico*. In the docked structure, while the NADPH molecule could be well aligned to the NADP moiety of NADP-DHF in the crystal structure, testosterone is positioned more deeply into the cavity compared to DHF with its ring structure stacking parallelly to the nicotinamide ring of NADPH (Fig. 3c). This is likely due to the absence of the hydrogen bond between E57^TM2^ and the N-4 group of DHF. As a result, the C-3 carbonyl group of testosterone forms hydrogen bonds with both E57^TM2^ and Y91^TM3^ and the 4-pro-(*R*)-hydride of NADPH is close to the Δ^4,5^ bond of testosterone (~2.5 Å) (Fig. 3c). Previous structural studies on the soluble steroid reductase AKR1D1 have shown that the steroid C-3 carbonyl formed hydrogen bonds with residues E120 and Y58. In ARK1D1, Y58 has been suggested to function as the general acid-base catalytic group to polarize the steroid C-3 carbonyl group together with E120 to facilitate hydride transfer and substrate enolization^34-36^. We propose that SRD5α2 employs a similar catalytic mechanism, in which residues E57^TM2^ and Y91^TM3^ polarize the C-3 carbonyl of testosterone by hydrogen bonding to facilitate the hydride transfer from NADPH to the C-5 atom of testosterone, leading to the formation of an enolized intermediate followed by reduction of the Δ^4,5^ bond in testosterone (Fig. 3d). Consistently, the optimum pH for the SRD5α2 catalytic activity is acidic (pH~5), which favors the protonation of E57^TM2^ in order to form a hydrogen bond with the C-3 carbonyl of testosterone^26^. Our mutagenesis studies showed that substitution of E57^TM2^ to a less acidic glutamine residue could compromise enzyme activity (Fig. 3e and Extended Data Fig. 4h). Likewise, the Y91F mutation, which eliminates the potential hydrogen bonding of Y91^TM3^ to testosterone, essentially abolished the conversion of testosterone to DHT by SRD5α2 in our experiments (Fig. 3e and Extended Data Fig. 4h), supporting our proposed catalytic mechanism (Fig. 3d).

Despite sharing a potentially similar catalytic mechanism, the relative orientations of the steroid substrates to NADPH are distinct between SRD5α2 and AKR1D1. In our crystal structure, the nicotinamide ring of NADP is oriented toward the α-face of DHF and residues E57^TM2^ and Y91^TM3^ are located on the other side of the core ring structure (Fig. 3a, c). In contrast, in AKR1D1, the nicotinamide ring of NADP^+^ is oriented toward the β-face of steroid substrates (Fig. 3f)^33-35^. Such a steroid-binding mode in SRD5α2 suggests that the hydride is transferred from NADPH to the C-5 atom of testosterone at the α-face, leading to the 5α-stereochemistry of DHT generated by SRD5αs.

### Structural dynamics of SRD5α2 for catalysis

The binding pocket of NADP-DHF only opens on the side of 7-TM, allowing the access of steroid substrates to SRD5α2 from the lipid bilayer (Fig. 2c). The cytosolic loops L1, L3 and L5 pack against each other to fully enclose the binding pocket for NADP (Fig. 1d, e), contrasting the highly exposed NADP^+^/NADPH-binding pockets in soluble AKRs and MaSR1^21,37^. All cytosolic loops are involved in the interactions with the adenine-ribose moiety of NADP. Such a conformation is compatible with the very tight binding of NADP-DHF to the enzyme^13^ while imposing a physical barrier for the NADPH/ NADP^+^ exchange during the reaction. It is unlikely that the nucleotides either enter or exit from the enzyme through the opening between TM1 and TM4 on the side of 7-TM considering the surrounding lipid environment and the highly polar nature of NADP^+^/NADPH (Fig. 2c). To lift the barrier, the cytosolic loops in SRD5α2 may undergo conformational changes during the reaction so that the cytosolic region can open up to expose the nucleotide-binding pocket to the cytosol before and after one reaction to allow the NADP^+^/NADPH exchange and thus efficient turnover of the reactions.

**Fig. 4:**
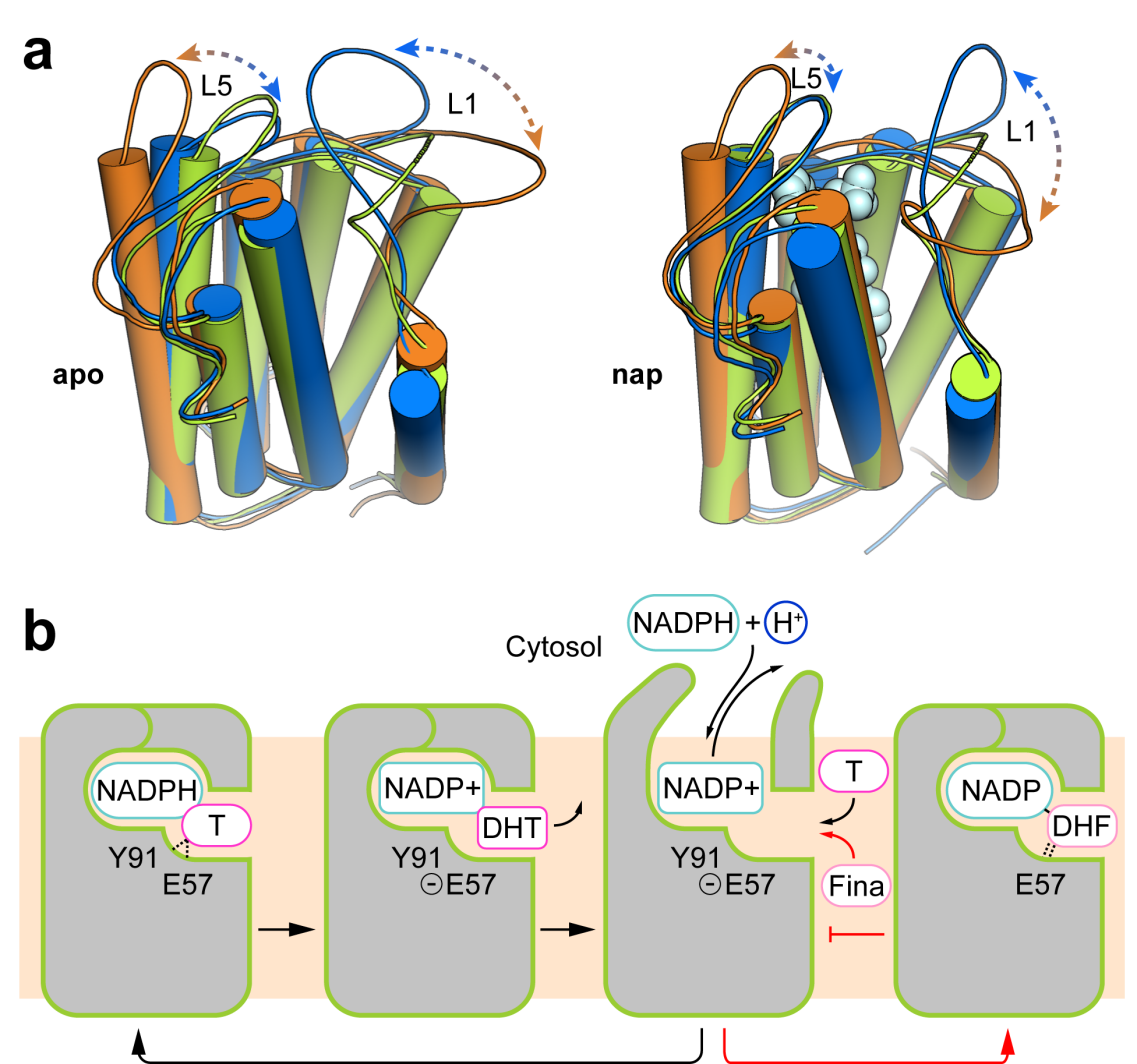
Dynamics of SRD5α2 during reaction. **(a)** Dominant conformational motions in the SRD5α2 MD simulations using principal component analysis (PCA). To visualize the motions, two extreme conformations from the principal components for the apo and nap states are indicated in orange and blue respectively. The SRD5α2 crystal structure colored in green is superimposed as a reference. PCA clearly indicates large-scale motions in L1 and L5, suggesting opening up of the nucleotide binding cavity. **(b)** Model for the dynamics of SRD5α2 in one cycle of reaction. The reaction can be inhibited by finasteride by forming a stable adduct with the NADPH cofactor to stabilize the closed conformation of SRD5α2. Testosterone and finasteride were indicated as “T” and “Fina”, respectively. The catalysis of testosterone and the finasteride inhibition are shown with black and red arrows, respectively.

To further investigate the conformational dynamics of SRD5α2, we performed molecular dynamics (MD) simulations of SRD5α2 with NADP^+^ (nap) and without NADP-DHF (apo) on microsecond timescales. Principle component analysis (PCA) of the MD trajectories clearly indicated high structural dynamics of the cytosolic loop L1 and to a lesser extent L5, which indeed resulted in the opening of the nucleotide-binding pocket during both nap and apo simulations (Fig. 4a and Extended Data Fig. 5a). In addition, in the apo simulations, L1 exhibited more pronounced conformational fluctuations as compared to it in the nap simulations (Extended Data Fig. 5a), presumably due to the stabilization of the cytosolic loops by NADP^+^ in the nap simulations (Fig. 2d and Extended Data Fig. 4a). Since L1 is involved in the binding pockets for both NADP and finasteride in the crystal structure, our simulation results suggest that L1 may function as a ‘gate’ domain to control the NADPH/NADP^+^ exchange and the binding of steroid substrates (Fig. 4b). Consistently, we observed higher B-factors in general for residues in L1 compared to residues in TMs and other cytosolic loops in the crystal structure, supporting the highly flexible feature of L1 (Extended Data Fig. 5b).

### Disease-related mutations of SRD5α2

**Fig. 5:**
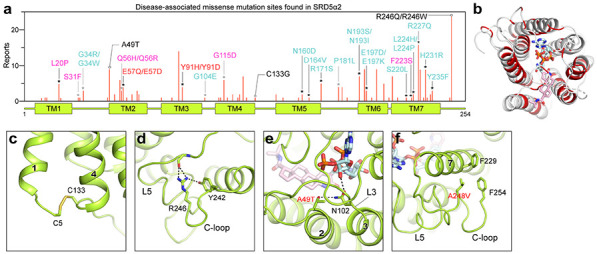
Structural analysis of disease-associated missense mutations of SRD5α2. **(a)** Distribution of identified missense mutations of SRD5α2. The red bar length for each mutation site indicates how many times it was reported based on the data collected from HGMD and literature. The sites that are involved in the binding of NADP-DHF and the formation of the binding cavity are indicated by black and grey asterisks respectively. The mutations presumably disrupting the steroid binding, the cofactor binding, and the catalysis are labeled in pink, cyan and red respectively. **(b)** Mapping of the mutation sites onto the SRD5α2 structure. The mutation sites are colored in red. **(c)-(d)** Environment of C133 and R246 suggesting their roles in protein folding. **(e)** Potential hydrogen-bonding interactions caused by the A49T mutation. **(f)** Potential hydrophobic interactions caused by the A248V mutation.

To date, over 100 genetic mutations on the *SRD5α2* gene have been identified according to the Human Gene Mutation Database (www.hgmd.cf.ac.uk) to cause the rare autosomal recessive disorder 5α-reductase deficiency^9,10^. Most mutations either abolish or reduce the activity of SRD5α2, leading to significantly reduced levels of DHT *in vivo*^10^. A majority of 5α-reductase deficiency-causing mutations are missense mutations that locate throughout the whole protein (Fig. 5a). By mapping reported missense mutations of SRD5α2 protein in our structure, we found that most of the mutation sites are at the ligand-binding cavity (Fig. 5b), suggesting that many of those mutations compromise the activity of SRD5α2 by impairing the cofactor/substrate binding or the catalytic process. Examples include two of the founder mutations for the 5α-reductase deficiency patients^10^, R227Q and R171S, which diminish the SRD5α2 activity likely by disrupting the hydrogen bonds with NADP (Extended Data Fig. 4c-e). Also supporting the critical roles of E57^TM2^ and Y91^TM3^ in our proposed catalytic mechanism, the mutations E57Q and Y91D have been shown to significantly reduce enzyme activity^26,38^. In addition, some mutations that are not in the ligand-binding cavity presumably impair protein folding/stability. For example, the C133G mutation eliminates the C5-C133 disulfide bridge that links TM1 and TM4, which may be important for the overall folding of SRD5α2 (Fig. 5c). As the most-frequently reported mutation site, R246^C^ in the C-loop forms multiple hydrogen bonds with residues in L5 (Fig. 5d), potentially stabilizing the L5 to be in place for the cofactor binding. Consistently, the R246W mutation showed a decreased NADPH-binding affinity in previous studies^9,26^.

On the other hand, two recurrent somatic mutations of SRD5α2, A49T and A248V, in human prostate cancer have been shown to significantly increase the activity of SRD5α2^29^. Especially, the A49T mutation has been linked to significantly increased risks of prostate cancer for certain populations^39^. A49^TM2^ is located at the cytosolic end of TM2 to face L3 and the adenine-ribose phosphate moiety of NADPH. The A49T mutation may lead to an additional hydrogen bond with residues from L3 to further stabilize the conformation of L3 (Fig. 5e). As for A248^C^, it tightly packs against L5. The A248V mutation likely results in additional hydrophobic interactions with nearby residues F229^TM7^ and F254^C^ to stabilize the C-loop, which in turn stabilizes the conformation of L5. Since both L3 and L5 are involved in the cofactor binding, the mutations of these residues may enhance the cofactor binding by stabilizing its binding pocket to increase the activity of SRD5α2.

## DISCUSSION

In contrast to the well-understood mechanisms for the function of soluble steroid reductases, for which numerous structures have been reported^37^, the molecular mechanisms governing the function of eukaryotic membrane-embedded steroid reductases have remained enigmatic due to limited structural information. To our knowledge, the reported structure of human SRD5α2 with the dualsteric ligand NADP-DHF represents the first structure of eukaryotic membrane-embedded steroid reductase. Together with computational studies, our structure unveils the binding cavity inside the 7-TM bundle for NADPH and steroid substrates with flexible cytosolic loops. Structural analysis and mutagenesis studies suggest the molecular mechanisms for enzyme catalysis and inhibition involving newly identified residues E57^TM2^ and Y91^TM3^. Because of the chemical differences in the steroid core ring, testosterone and finasteride adopt different binding poses relative to NADPH so that the Δ^4,5^ bond in testosterone and the Δ^1,2^ bond in finasteride can be reduced by SRD5α2 to generate distinct end products. Our results well explain the 5α-reduction reactions catalyzed by SRD5αs, contrasting the 5β-reduction reactions catalyzed by soluble steroid reductases. The mechanism-based irreversible action of finasteride and dutasteride led to their successful use as anti-androgen drugs, which may be repurposed for treating COVID-19 patients with excessive androgen receptor signaling ^18,19^. Mapping disease-related mutations of SRD5α2 to our structure also provides feasible molecular mechanisms for the effects of those mutations in the 5α-reductase deficiency and the prostate cancer.

**Fig. 6:**
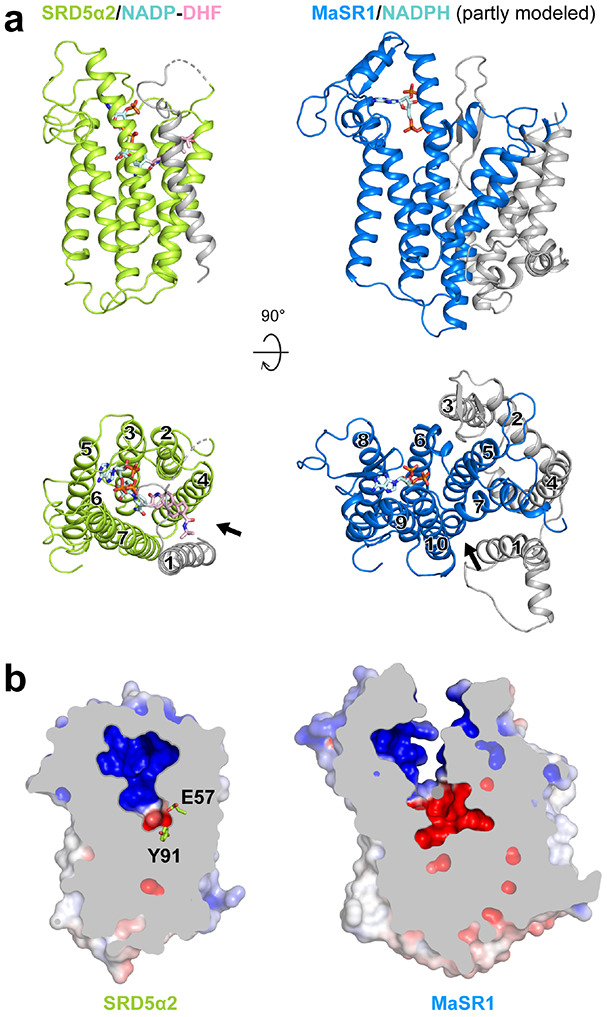
Structural comparison of SRD5α2 and MaSR1. **(a)** Structural superposition of the transmembrane regions of SRD5α2 and MaSR1. The core transmembrane regions in SRD5α2 and MaSR1 that can be structurally aligned were shown as colored ribbons while the rest parts were shown as grey ribbons. The potential substrate entry ports in SRD5α2 and MaSR1 were indicated by arrows. **(b)** Occluded and exposed ligand-binding cavities in SRD5α2 and MaSR1, respectively.

The SRD5α2 structure together with simulation studies reveal unexpected structural features for the binding of NADP^+^/NADPH and steroid substrates. The cytoplasmic loops L1, L3 and L5 enclose the binding pocket of NADPH inside the 7-TM bundle of SRD5α2 to position it close to testosterone for catalysis. To our knowledge, such a structure feature has not been observed in other enzymes using pyridine nucleotides including NADH and NADPH as cofactors. Our MD simulation results suggest that the cytoplasmic loop L1 undergoes dramatic conformational changes during the reaction to allow the NADP^+^/NADPH exchange (Fig. 4a). The potential large energy barrier for the cytosolic region to overcome to open up the NADPH-binding pocket may hinder NADPH binding. As a result, the reported dissociation constant of NADPH for SRD5α2 (~3-10μM)^1^ is higher than that of NADPH for the soluble steroid reductase AKR1D1 with a highly exposed NADPH-binding pocket (~0.5μM)^35^. As for the steroid substrates, they likely access the ligand-binding pocket of SRD5α2 from the lipid bilayer through the opening between TM1 and TM4 (Fig. 2c, 6a), which is analogue to the lateral ligand entrance mechanism for several GPCRs with lipid ligands^40,41^.

Despite sharing very little sequence similarity and having different structural topology, human SRD5α2 and bacterial MaSR1 can be aligned in their core structure of six transmembrane helices (TM2-7 in SRD5α2) that participates in the binding of NADPH and substrates (Fig. 6a). There is also an opening between TM7 and TM10 in MaSR1 (Fig. 6a), which may serve as the potential entry port for its substrates. Such conserved structural features imply that these two enzymes and likely other membrane-embedded steroid reductases take NADPH from the cytosol through cytoplasmic loops and steroid substrates from the lipid bilayer into ligand-binding cavities inside the cell membrane for catalysis. In contrast to the buried NADP in SRD5α2, the partially modeled NADPH in the structure of MaSR1 occupies a different binding pocket that is exposed to the cytosol (Fig. 6a, b)^21^, suggesting diverse NADPH recognition mechanisms for membrane-embedded steroid reductases. We speculate that these enzymes are likely to adopt distinct structural features within their TM bundles so NADPH can be appropriately positioned towards their steroid substrates for site-specific carbon-carbon double bond reduction. Further structural investigation on other important eukaryotic membrane-embedded steroid reductases including SRD5α3 and DHCR7 is needed to understand the potentially different mechanisms by which these enzymes reduce specific carbon-carbon double bonds at different positions of chemically similar steroid substrates using NADPH.

## Methods

### SRD5α2 expression and purification

cDNA of human full-length wild-type SRD5α2 was synthesized (IDT) and cloned into pFastBac vector with an N-terminal signal peptide followed by a Flag epitope and an 8xhistidine tag. One tobacco-etch virus (TEV) protease cleavage was introduced after the His tag. SRD5α2 protein was expressed in the insect Sf9 cells using the Bac-to-Bac baculovirus system (ThermoFisher). Cells were infected by baculovirus at a density of 4.0 x 10^6^ cells/mL and harvested after 48 h at 27 °C. To stabilize the protein, all purification steps were accomplished in the presence of the inhibitor finasteride (Tocris). Sf9 cells were lysed in buffer containing 20 mM Tris pH7.5, 2.0 mg ml^−1^ iodoacetamide, 0.2 μg ml^−1^ leupeptin, 100 μg ml^−1^ benzamidine and 0.5 μM finasteride. The cell pellet was further resuspended and homogenized using the glass dounce homogenizer in buffer containing 20 mM HEPES pH7.5, 750 mM NaCl, 1.0 μM finasteride, 20% glycerol, 1% dodecyl maltoside (DDM), 0.1% cholesterol hemisuccinate (CHS), 0.2% sodium cholate, 2.0 mg ml^−1^ iodoacetamide, 0.2 μg ml^−1^ leupeptin, 100 μg ml^−1^ benzamidine and salt active nuclease (ArcticZymes) and incubated at 4°C for 90 min. After centrifugation, the supernatant was collected and incubated with Ni-NTA resin (GE healthcare) in the presence of 8 mM imidazole to prevent the non-specific binding at 4°C overnight. The resin was pelleted and washed with 20 mM HEPES pH7.5, 500 mM NaCl, 1.0 μM finasteride, 0.1% DDM, 0.02% CHS and 40 mM imidazole. The bound protein was eluted using the same buffer with 400 mM imidazole and then loaded onto the anti-Flag M1 affinity column after supplementing with 2 mM CaCl_2_. After extensively and slowly exchanging detergent to 0.1% lauryl maltose neopentyl glycol (LMNG) (Anatrace), the protein was eluted using buffer containing 20 mM HEPES pH7.5, 100 mM NaCl, 1.0 μM finasteride, 0.01% LMNG, 0.001% CHS, 200 μg ml^−1^ synthesized Flag peptide (GL Biochem) and 5 mM EDTA. TEV protease and PNGase F were added to the eluted protein and incubated at 4°C overnight. The treated sample was reloaded onto Ni-NTA column to remove TEV protease and the flow-through fraction was collected. The protein was further purified by size-exclusion chromatography using a Superdex S200 increase column (GE healthcare) in buffer containing 20 mM HEPES pH7.5, 100 mM NaCl, 1.0 μM finasteride, 0.01% LMNG and 0.001% CHS. The monodispersed fractions were pooled together and concentrated to 50 mg ml^−1^ for crystallization.

### Crystallization

Purified protein in complex with finasteride was reconstituted into the lipidic cubic phase (LCP)^22^ through mixing protein with monoolein/cholesterol lipid mixture (10:1 w/w) (Avanti) at a weight ratio of 2:3 (protein: lipid) using coupled syringes (ArtRobbins). Using a Gryphon LCP robot (ArtRobbins), the LCP mixture was dispensed onto 96-well glass sandwich plates in 20 nL drops and then overlaid with 700 nL precipitant conditions. Plates were incubated at 20 °C and crystals grew in the condition of 100 mM tris-sodium citrate pH5.0, 28-34% PEG600, 100-150 mM NaCl and 100 mM Li_2_SO_4_. Crystals were collected from the LCP matrix and frozen in liquid nitrogen for data collection.

### *De novo* structure modeling

To predict the 3D model of human SRD5α2, several alternative multi-sequence alignments were generated using a diverse set of sequence databases under different sequence search engines ^42^ and then fused by implementing a deep residual network with a *strip pooling* module ^43^ to effectively capture long-range relationship of residual pairs. Following trRosetta ^44^, we generated the 3D model of SRD5α2 from the predicted distance and orientation using constrained minimization. Specifically, the predicted distance- and orientation-probabilities were first converted into potentials, which are then used as restraints to be fed into Rosetta together with coarse-grained energy optimization. Finally, the top 5 folded structures satisfying the restraints were selected according to Rosetta energy as initial models for structure determination.

### X-ray data collection and structure determination

X-ray diffraction data was collected at the beamline 23ID-B, GM/CA of Advanced Photon Source (APS) in the Argonne National Laboratory at Chicago. Each crystal was exposed with a 10 μm x 10 μm beam for 0.2 s and 0.2-degree oscillation per frame to collect 20 degrees of rotation data. Data sets from 5 crystals were processed and merged in HKL2000 software ^45^.

To determine the phase of the crystal structure, the *de novo* structure models were used as the search models for molecular replacement. One of the models was successfully used for searching the solution by Molrep in CCP4 package ^46^. The initial phase was largely improved by using density modification methods in PHENIX ^47^. COOT ^48^ was used for model rebuilding based on the improved electron density. The rebuilt model was further refined in PHENIX with an additional TLS refinement was performed. The model quality was check by MolProbity ^49^. The final refinement statistics are listed in Table 1. All structure figures were prepared by PyMOL (http://www.pymol.org/).

### Molecular docking

The NADP-DHF adduct from the crystal structure was removed and an analogous NADP-testosterone adduct with NADP covalently bound to the C-5 atom of testosterone at the α-face was docked to the crystal structure. In the docked pose, the covalent link was removed to obtain NADPH and testosterone. This was followed by optimizing the ligands and their surrounding 5 Å protein residues. Thereafter, NADPH was redocked to the optimized protein, followed by redocking testosterone to the NADPH docked protein. The docking poses were filtered to be within 10 Å RMSD of the X-ray ligand densities to obtain the pose for NADPH and testosterone docked to SRD5α2. Docking calculations were performed using Glide^50^.

### Molecular modelling and MD simulations

SRD5α2 models were built based on the solved crystal structure to incorporate unresolved residues 1-4 (N-terminus) and 39-43 (loop L1) using Modeller ^51^. We generated 5000 models and selected top 10 scoring models based on DOPE scoring function ^52^. From the top 10 models, two most structurally diverse protein configurations were selected to start molecular dynamics (MD) simulations for unliganded (apo0, apo1) and NADP^+^-bound (nap0, nap1) states. NADP^+^ was placed at the NADP density in the crystal structure. NADP^+^ was modelled at a molecular charge of −2. Ionization states of protein residues were calculated at *p*H=7 by solving Poisson-Boltzmann equation in continuum electrostatics models (ε_protein_=20; ε_solvent_=80) using APBS ^53^. All residues were found to be in their standard ionization states. Based on the crystal structure, a disulfide bond was modelled between Cys-5 and Cys-133. The protein models were embedded in a pre-equilibrated 1-palmitoyl-2-oleoyl-sn-glycero-3-phosphocholine (POPC) lipid bilayer followed by solvation (TIP3P water model) and neutralization using potassium and chloride ions at 150 mM. The simulation setups comprised *ca*. 44000 atoms each. CHARMM36 forcefield was employed for the MD simulations including protein, ligands, lipids, water and ions ^54^. The systems were first energy minimized for 10000 steps and then heated gradually from 0 K to 310 K for 250 ps using a Langevin thermostat with heavy atoms restrained at 10 kcal mol^−1^ Å^−2^ in an *NVT* ensemble. The heated systems were subjected to 8 successive rounds of 1 ns equilibration steps. During the equilibration, protein and ligand heavy atoms were subjected to harmonic restraints and lipids were subjected to planar restraints to maintain bilayer planarity. The harmonic restraints for each step were relaxed progressively going from 10 kcal mol^−2^ Å^−2^ to 0.1 kcal mol^−1^ Å^−2^. The equilibrations were performed at a 1 fs timestep at *T* = 310 K and *P* = 1 bar using the Langevin thermostat and Nosé-Hoover Langevin barostat in *NPT* ensemble. The production runs were performed with a hydrogen-mass repartitioning (HMR) scheme with a timestep of 3.6 fs with a non-bonded cutoff at 12 Å ^55^. Long range electrostatics were evaluated with the Particle Mesh Ewald method. Protein and lipid bond-lengths were constrained with the SHAKE algorithm. Each system was simulated for *ca*. 1.25 μs, giving a total simulation time of ca. 5.4 μs (2.7 μs apo and 2.7 μs nap). Trajectory snapshots were saved at every 50 ps. The simulations were performed with NAMD 2.13 ^56^. The simulation setup was constructed using CHAMM-GUI^57^. In order to analyze the multidimensional conformational landscape, we performed principal component analysis (PCA) of MD trajectories to identify the dominant modes of protein motions (principal components) in the apo and nap simulation states. PCA was performed with pyPcazip ^58^. Visual Molecular Dynamics (VMD) ^59^ was employed for visualization and for performing RMSF analysis to probe protein mobility.

### Enzyme activity measurement

We used insect cell membranes overexpressing different constructs of SRD5α2 in the measurement of enzyme activity. Sf9 cells expressing wild type SRD5α2 (WT) and mutants E57Q and Y91F were harvested after 48 h transfection. The expression level of each construct was determined by flow cytometry using FITC-labeled anti-FLAG M2 antibody (Sigma Aldrich) in the presence of 0.5% tritonX-100. For membrane preparation, the cell pellets were resuspended in lysis buffer containing 20 mM Tris pH7.5, 1 mM EDTA, 0.2 μg ml^−1^ leupeptin and 100 μg ml^−1^ benzamidine. The lysed and homogenized samples were centrifuged at 1,200xg for 8 min. The supernatant was further centrifugated at 300,000xg for 50 min to get the membrane pellets, which were resuspended in buffer containing 20 mM HEPES pH7.5, 100mM NaCl, 1 mM EDTA, 0.2 μg ml^−1^ leupeptin and 100 μg ml^−1^ benzamidine and stored at −80°C.

The enzyme activity was assayed using the prepared membrane fractions (0.3 mg each reaction) in the presence of 0.5 mM NADPH and 0.5 mM testosterone in 0.1 M sodium-citrate pH 5.0 buffer. Finasteride at 0.5 mM was used as a negative control. After incubation for 4 hours at 37°C, the steroids were extracted with chloroform, evaporated by centrifugal concentrator and re-dissolved in methanol for liquid chromatography–mass spectrometry (LC-MS) analysis using buffer A containing 1 mM ammonium formate and buffer B containing 100% methanol. The ratios of the peak area of dihydrotestosterone to the peak area of testosterone were calculated as indicators of enzyme activity. The data were processed by Prism8 (GraphPad). The experiments on the wt SRD5α2 (WT) were repeated 5 times. The experiments on WT with 0.5 mM finasteride were repeated 4 times. The experiments on each mutant, E57Q or Y91F, were repeated 3 times. The data were represented as mean ± SEM.

### Data availability

The coordinates and structure factors have been deposited in the Protein Data Bank under the accession codes PDB 7BW1. Distribution of research materials generated in this study including plasmids will need appropriate Material Transfer Agreements (MTAs).

## Supplementary Material

Supplement

## Figures and Tables

**TABLE 1.** X-ray data collection and refinement statistics.

Data collection	
Space group	*P* 6 2 2
Cell dimensions	
*a, b, c* (Å)	107.449, 107.449, 103.372
α, β, γ (°)	90, 90, 120
Resolution (Å)	50–2.8 (2.85–2.8)
*R*_merge_^a^	0.248 (1.274)
*I/σI*	13.0 (1.05)
*CC*_1/2_^b^	1.0 (0.415)
Completeness (%)	99.7 (100.0)
Redundancy	9.6 (6.8)
Refinement	
Resolution (Å)	50–2.8 (3.19–2.8)
No. reflections	9155 (2857)
*R*_work_ / *R*_free_^c^	0.239 (0.297) / 0.265 (0.336)
No. atoms	2036
Protein	1926
Ligand	110
Mean *B* (Å)	75.8
r.m.s. deviations	
Bond lengths (Å)	0.003
Bond angles (°)	0.77
Ramachandran analysis	
Favored region (%)	95.9
Allowed region (%)	4.1
Outliers (%)	0

The numbers in parentheses represent values for the highest resolution shell.

a R_merge_ = Σ∣I_i_ - I_m_∣/ΣI_i_, where Ii is the intensity of the measured reflection and I_m_ is the mean intensity

b *CC*_1/2_ is the correlation coefficient of the half datasets.

c R_work_ = Σ∥F_obs_∣ - ∣F_calc_∥/Σ∣F_obs_∣, where F_obs_ and F_calc_ are observed and calculated structure factors.

R_free_ = Σ_T_∥F_obs_∣ - ∣F_calc_∥/Σ_T_∣F_obs_∣, where T is a test data set of about 5 % of the total reflections
