# Supplementary material for: Structure of human steroid 5α-reductase 2 with anti-androgen drug finasteride
Source: Res Sq. 2020 Jul 15:rs.3.rs-40159. Preprint. [Version 1] doi: 10.21203/rs.3.rs-40159/v1 (PMC7373137; doi:10.21203/rs.3.rs-40159/v1)
Supplement: Supplement [file S5A2ExtendedDataFigures.pdf]

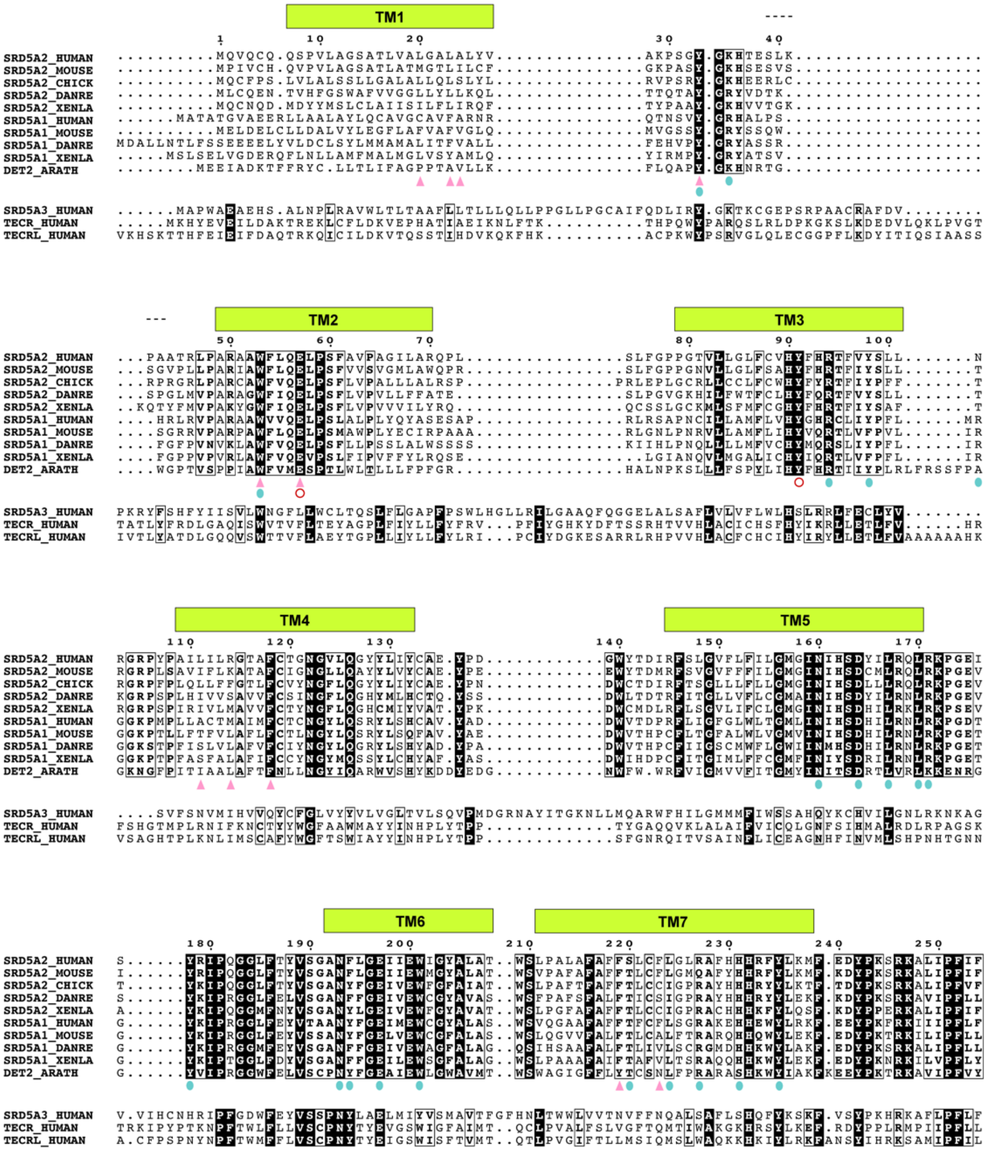

Extended Data Figure 1. Multi-sequence alignments of SRD5α enzymes.

The sequences of the SRD5α1/2 subfamily members across different species from human to plant were aligned first. The other SRD5α family members in human containing SRD5α3, GSPN2 (TECR), and GSPN2-like (TECRL) were then aligned to the first alignment. The species were represented by "DANRE" for *Danio rerio* and "XENLA" for *Xenopus laevis*. DET2 is the SRD5α1/2 homologue in *Arabidopsis thaliana*. The transmembrane helical regions are labeled above the alignment. Residues that are identical and highly similar in each alignment are shown in black and white boxes, respectively. The residues involved in the DHF-binding and NADP-binding are indicated by pink triangles and cyan circles, respectively. The two residues involved in the catalysis are indicated by open circles.

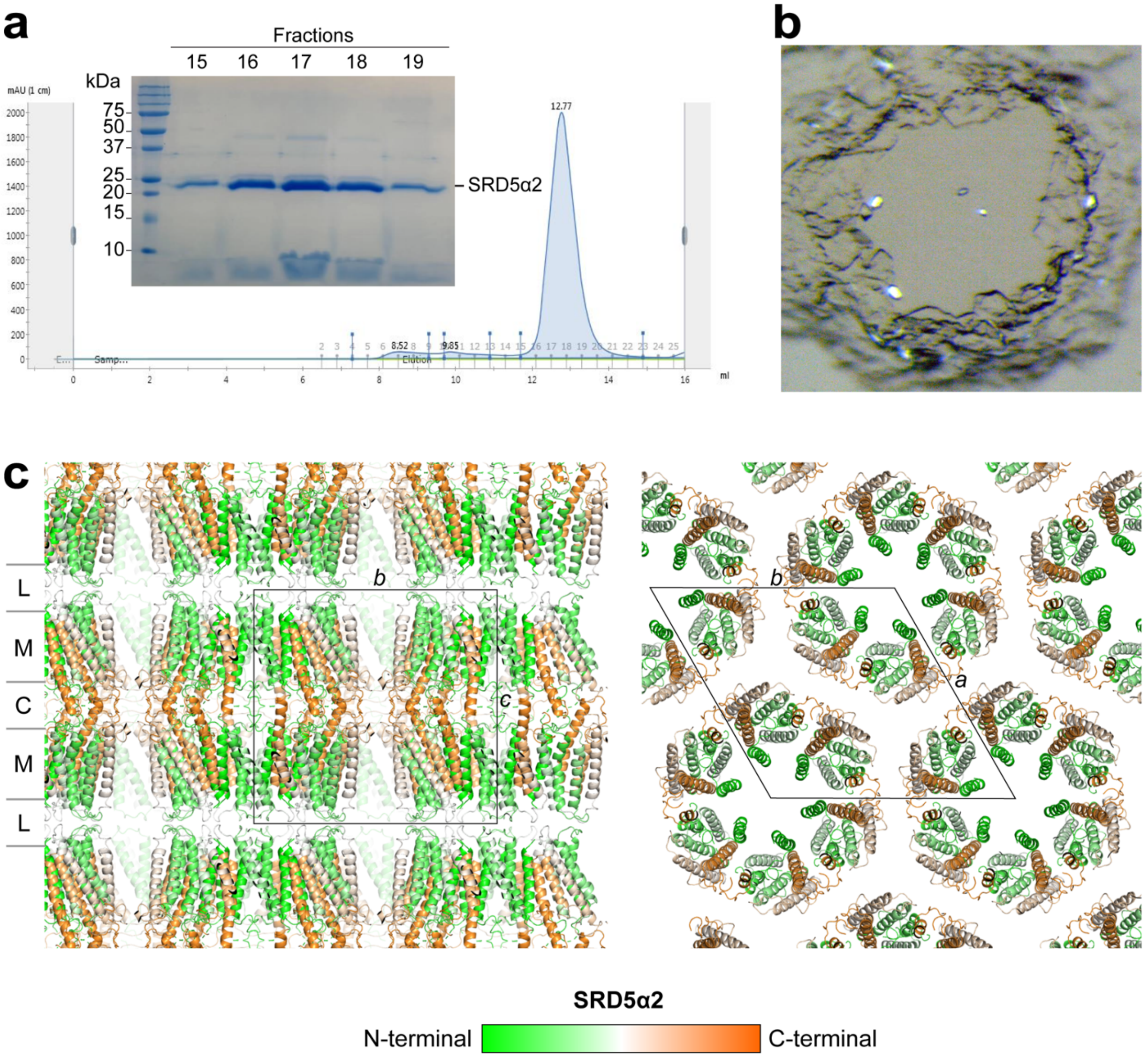

Extended Data Figure 2. SRD5α2 purification and crystallization.

(a) Purification of SRD5α2 by size-exclusion chromatography (SEC). The protein homogeneity was high as indicated by the monodispersed peak in SEC and the following SDS-PAGE analysis. (**b**) Crystals of SRD5α2 in the lipid mesophase. (**c**) Two views of crystal packings in the SRD5α2 crystal. A unit cell was indicated. It is interesting to note the *P622* space group of the SRD5α2 crystals is very uncommon for membrane protein crystals from the lipid mesophase. SRD5α2 forms crystallographic hexamers on each lipid bilayer. The cytosolic region, transmembrane region, and lumenal region of SRD5α2 in the crystal packings are indicated as “C”, “M”, and “L”, respectively.

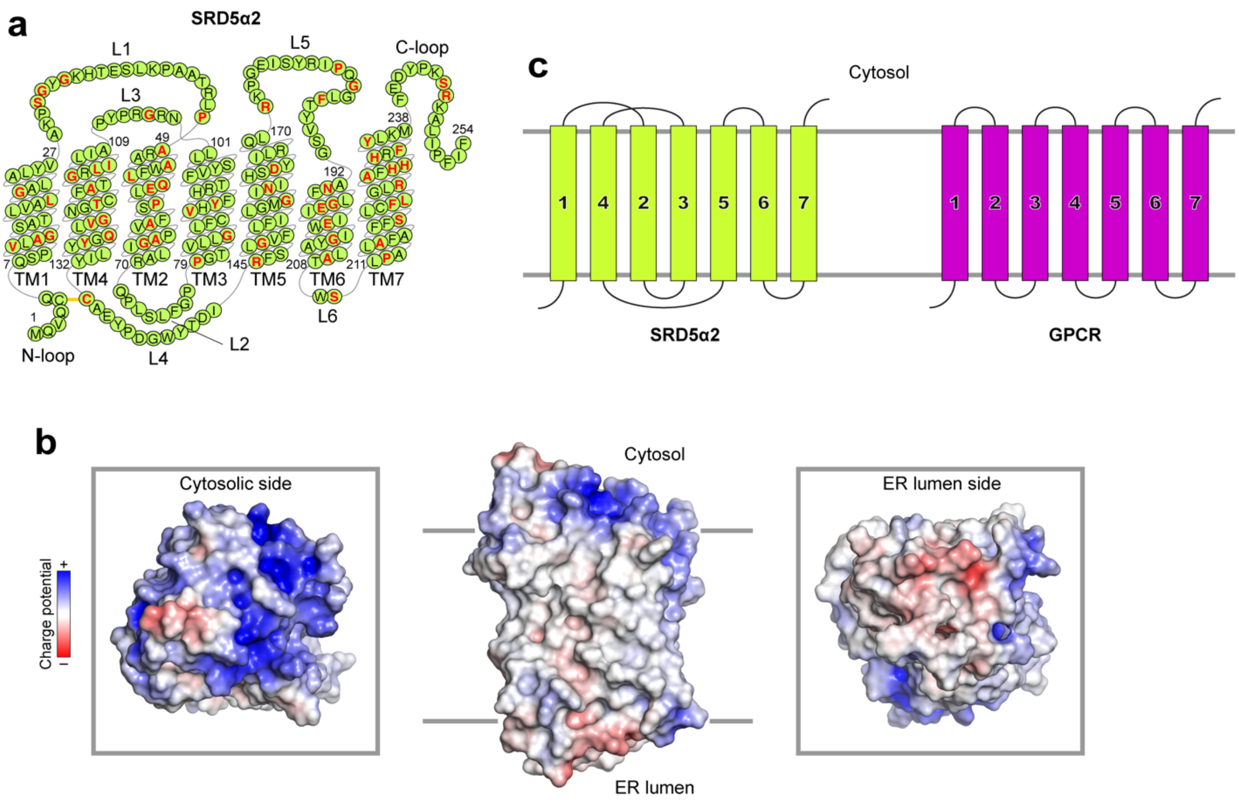

Extended Data Figure 3. Topological analysis of SRD5α2.

(a) The amino acid sequence of SRD5α2 in different TM and loop regions. The disease-associated missense/nonsense mutation sites were colored in red. The C5-C133 disulfide bridge was indicated by a connecting line between the two cysteine residues. **(b)** Surface charge potential analysis of SRD5α2. Positively charged regions indicates the cytosolic side. **(c)** The different topological arrangement of the seven TMs in SRD5α2 and GPCRs.

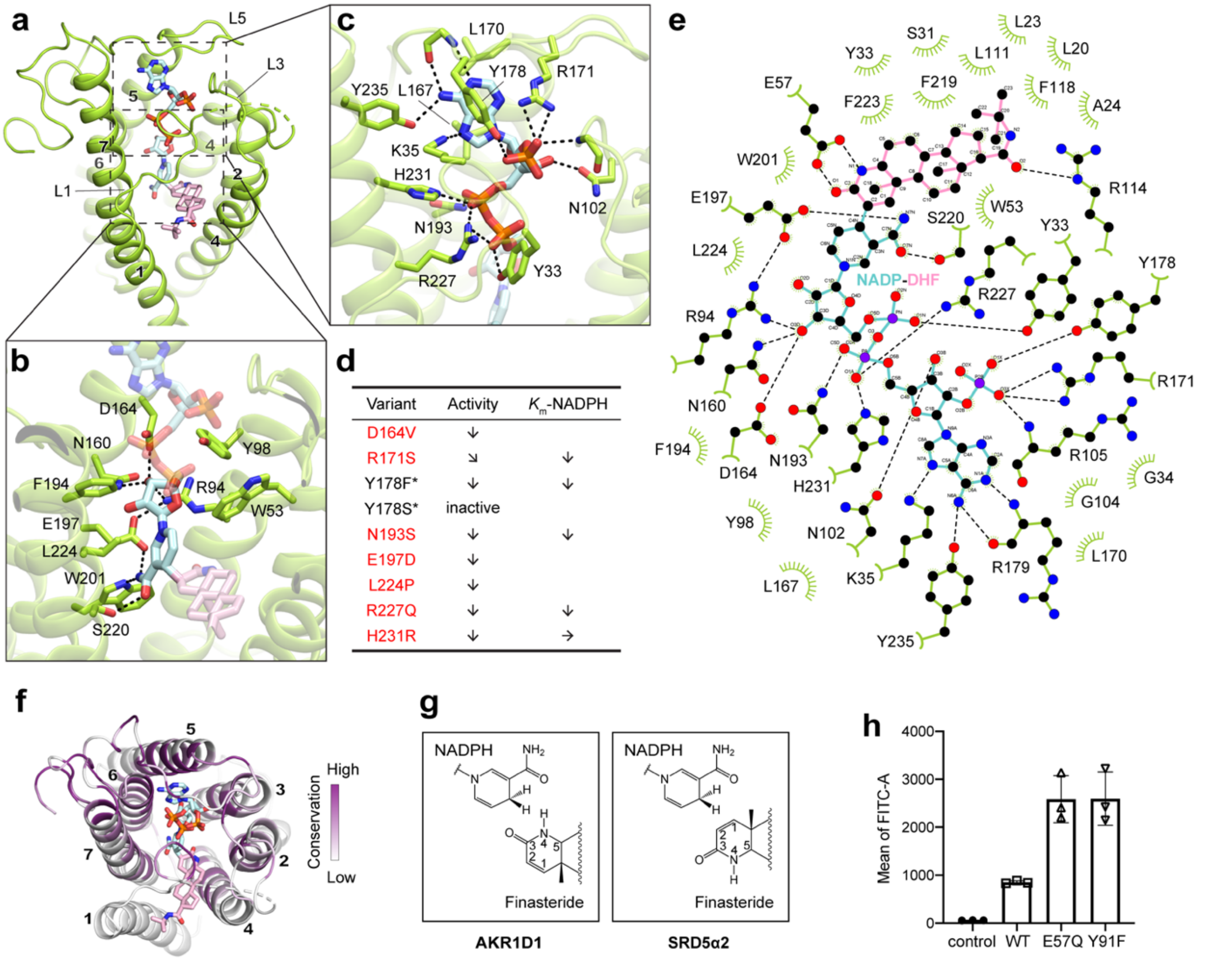

Extended Data Figure 4. Molecular details of ligand-binding cavity, finasteride inhibition and mutagenesis data.

(a) The overall view of the NADP-binding pocket. **(b)** The detailed interactions between the nicotinamide-ribose moiety of NADP and SRD5α2. **(c)** The detailed interactions between the diphosphate moiety of NADP and SRD5α2. **(d)** The NADP-DHF/SRD5α2 interaction analysis. The polar and hydrophobic interactions were shown as a 2D view. **(e)** Summary of previous enzymologically studied mutations in the NADP-binding interface. The mutations found in patients with steroid 5α-reductase deficiency were colored in red. The disruptive effects of the Y178F and Y178S mutations were derived from the mutagenesis study of rat SRD5α2. **(f)** Sequence conservation analysis on SRD5α1 and SRD5α2. The conservation is derived from the sequence alignment of SRD5α1 and SRD5α2 as shown in Figure S1. (**g**) Different orientations of finasteride relative to NADPH in AKR1D1 and SRD5α2 leading to different inhibition mechanisms. (**h**) Cell surface expression levels of SRD5α2 and its two mutants. The membrane fractions from these cells were used for measuring enzymatic activities as shown in Fig. 3E. Expression levels were determined by fluorescent antibody staining of Sf9 cells. All data are presented as mean ± SEM of 3-4 independent experiments.

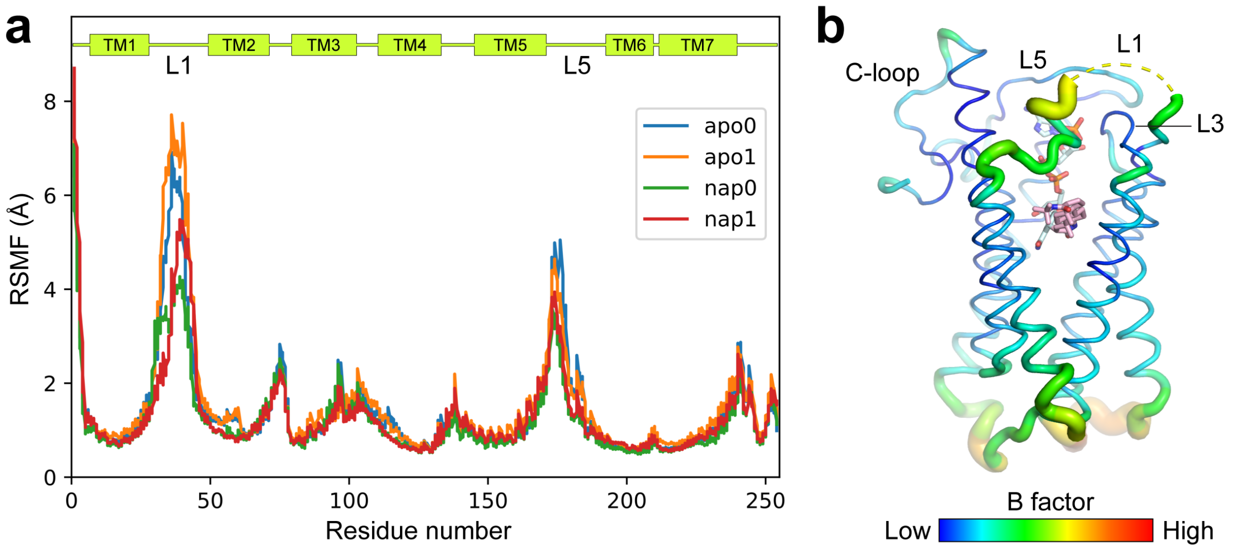

Extended Data Figure 5. The dynamic feature of the cytosolic loops in SRD5α2.

(a) Root-Mean-Square-Fluctuation (RMSF) plots of the protein calculated from the two apo and two nap simulation states. The cytosolic loops, L1 and L5 show the largest fluctuation in simulation. (**b**) The B-factor putty view of the SRD5α2 structure showing the relatively higher B-factors in the L1 region.
